# Development of a Plate Linear Ultrasonic Motor Using the Power Flow Method

**DOI:** 10.3390/mi15081016

**Published:** 2024-08-08

**Authors:** Yue Jian, Zhen Liu, Junfeng He, Wenjie Zhou, Huazhuo Liang

**Affiliations:** 1 School of Mechatronic Engineering, Guangdong Polytechnic Normal University, Guangzhou 510665, China; jianyue@gpnu.edu.cn (Y.J.); hejunfeng@gpnu.edu.cn (J.H.); zhouwj1001@163.com (W.Z.); lianghuazhuo@gpnu.edu.cn (H.L.); 2 Jihua Laboratory, No. 28 Island Ring South Road, Guicheng Street, Nanhai Distrct, Foshan 528200, China

**Keywords:** linear ultrasonic motor, optimization method, large thrust, narrow space

## Abstract

Linear ultrasonic motors can output large thrust stably in a narrow space. In this paper, a plate linear ultrasonic motor is studied. Firstly, the configuration and operating principle of the Π-type linear ultrasonic motor is illustrated. Then, two slotting schemes are put forward for the stator to enlarge the amplitude of the driving foot and improve the output performance of motor. After that, a novel optimization method based on the power flow method is suggested to describe the energy flow of stator, so as to estimate the slotting schemes. Finally, the prototypes are manufactured and tested. The experimental results show that the output performance of both new motors are excellent. The maximum output thrust of the arc slotted motor is 76 N/94 N, and the corresponding maximum no-load speed is 283 mm/s/213 mm/s, while the maximum output thrust of V-slotted motor reaches 90 N/120 N, and the maximum no-load speed reaches 223 mm/s/368 mm/s.

## 1. Introduction

A linear ultrasonic motor (LUSM) is a novel type of actuator that utilizes the inverse piezoelectric effect of piezoelectric ceramics and the ultrasonic vibration of an elastic body [1,2,3,4,5]. To date, they are becoming widely used in various fields, especially in narrow spaces, thanks to their compact structure, quick response, no electromagnetic interference, and other advantages [6,7,8,9]. However, the shortcomings of output characteristics and operation stability of existing motors limit their application. A LUSM with both small structure and large output thrust has always been the research hotspot in this field [10,11,12,13].

In 1998, Kurosawa proposed a standing-wave LUSM, composed of two mutually perpendicular Langevin transducers [14]. This V-type structure is beneficial for delivering the energy of longitudinal vibration to the driving foot so that the motor achieves a good output performance. The volume of this stator is 86 mm × 86 mm × 20 mm. Experiments showed that the output thrust reached 51 N and the output velocity reached 3.5 m/s. Thus, standing-wave LUSMs have attracted extensive attention. Scholars have proposed a variety of novel LUSMs for different occasions. In 2001, Yun C put forward a high power LUSM, which achieved 92 N output thrust with the volume of stator of 92 mm × 40 mm × 40 mm [15]. However, these LUSMs with large output thrust also have a relatively large volume, which is not suitable for a narrow space.

Due to its flat structure and high space utilization, the LUSM plate type is especially suitable to be the driving element with certain thrusts in a narrow installation space [16,17,18]. In 1989, Tomikawa proposed a LUSM with a rectangular plate structure, which uses the longitudinal and bending vibration of a rectangular plate at the same frequency to form elliptical motion at the driving foot and drive the motion of the slider [19]. Since then, some scholars have studied plate LUSMs. However, compared with the ultrasonic motors composed of Langevin oscillators, the output performance of plate-type LUSMs is low. Moreover, at present, most plate-type LUSMs use longitudinal and bending coupled modes of the plate to excite the vibration of the stator. Among these, the most famous ones are the L1B2 motors, which have attracted the attention of many researchers [20,21]. However, the coupled modes excitation method increases the difficulty of design process and is not conducive to the miniaturization of the stator. The single mode excitation method can effectively solve this problem [22,23,24]. In 2018, a Π-type linear ultrasonic motor driven by a single mode was proposed [25]. Compared with the existing linear ultrasonic motors, the novel motor with a plate structure can effectively output a large thrust in a narrow space. Experiments showed that the maximum output thrust of this motor is up to 110 N, and the maximum no-load speed is 273 mm/s, with a stator volume of 80 mm × 62 mm × 8 mm.

In this paper, focused on linear ultrasonic motors, which can output large thrust stably in a narrow space, two novel plate linear ultrasonic motors are put forward. First, the configuration and operating principle of a Π-type linear ultrasonic motor is illustrated. Then, in order to further improve the output performance, two slotting methods are proposed that can effectively reduce stiffness of head blocks and enlarge the amplitudes of driving feet. After that, a novel optimization method for ultrasonic motors based on the power flow method is suggested to evaluate the energy flow of the stator. At last, prototypes are manufactured and their output performances are evaluated through experiments.

## 2. Structural Design

### 2.1. Working Principle of Motor

**Figure 1:**
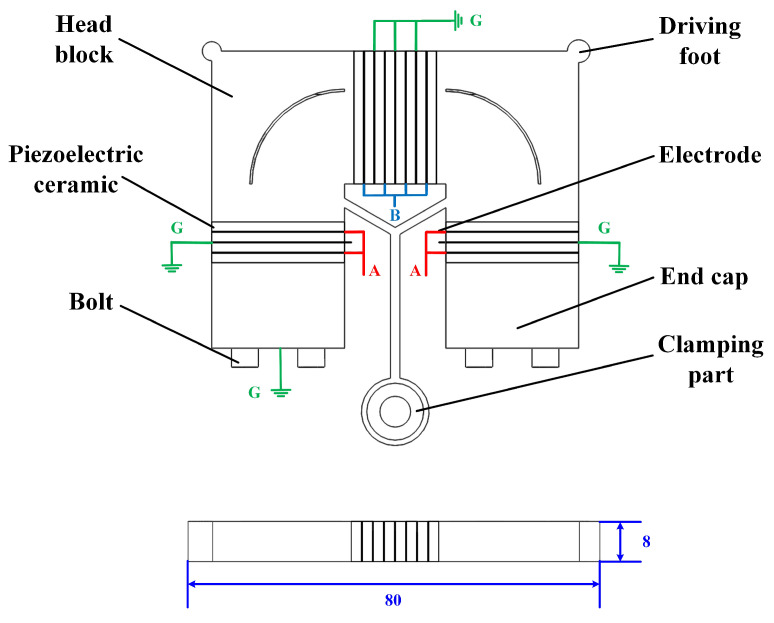
Specific view of the stator.

The view of the stator is shown in Figure 1. As a whole, a plate with a length–height ratio of 10 is adopted, so the stator can be placed in a narrow space.The dimension of the stator (excluding the clamping part) is 80 mm × 62 mm × 8 mm. Specifically, the stator is arranged as an axially symmetrical Π-type structure, with two driving feet sitting on the vertices of both sides, and a clamping part occupies the middle of the stator. The stator also includes head blocks, piezoelectric ceramics, electrodes, end caps, and bolts. Among them, The head blocks and end caps are fabricated with Cr12 due to its favorable hardness and wear-resisting properties. The piezoelectric ceramics (PZT-8) are fastened between the elastomers through bolts. The material parameters of the Π-type motor are shown in Table 1. Compared with most existing plate-type LUSMs with piezoelectric ceramics stuck to the elastomers, this sandwich-type connection utilizes the $d_{33}$ effect with the higher electromechanical coupling coefficient of piezoelectric ceramics, which can effectively improve the energy conversion efficiency and output performance of motor [26,27].

**Figure 2:**
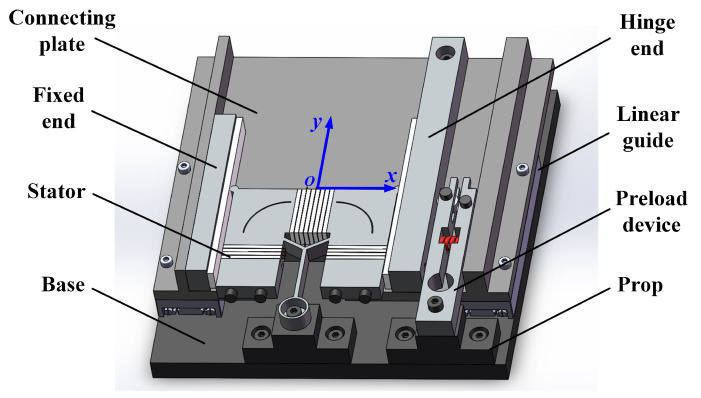
The integrated structure of the motor.

The integrated structure of a motor, as well as the overall structure of a motion platform driven by linear ultrasonic motor, is presented in Figure 2. The stator and the preload device are fixed on the base. Two linear guides are used as mobile pairs of this motion platform. Their rails are installed on two sides of the base, while their slide blocks are connected by a plate. The connecting plate and two slide blocks compose the slider of the motor. A high rigidity friction ceramic strip made of ${\mathrm{Al}}_{2}$$O_{3}$ is bonded to one side of the slider to improve contact performance and reduce wear losses caused by frictional coupling between the driving foot and the friction ceramic. When the stator is excited to work, it can drive the slider to move in both positive and negative directions along the Y-axis. The motion of the slider in the forward direction is indicated as the outward motion, while the motion in the opposite direction is considered inward motion.

**Figure 3:**
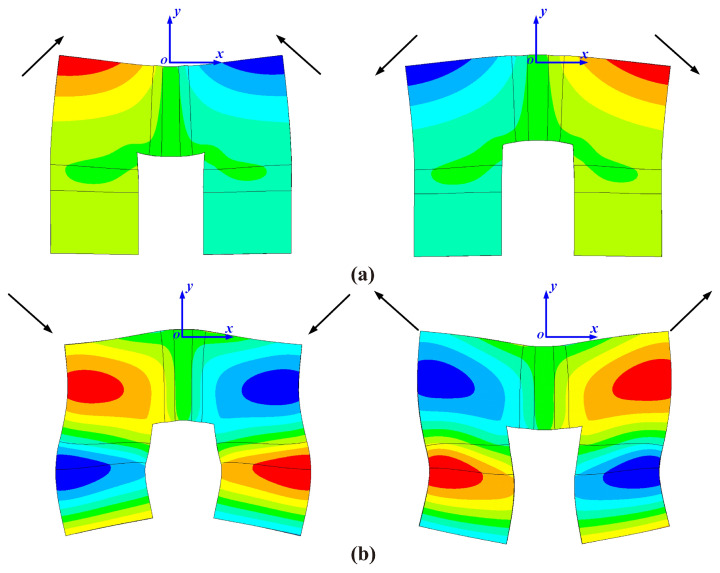
The working principle of the Π-type motor for (**a**) inward motion and (**b**) outward motion.

For a Π-type motor, the motions in two directions are completed directly by different modes. There is no interference between them and no mode superposition is needed. Compared with coupled mode LUSMs, the design process is simplified and the miniaturization is easier. The specific working principle of the motor is shown in Figure 3.

If the oscillator that is composed of a head block, piezoelectric ceramics, and another head block in sequence along the X-direction is considered as a horizontal rectangular vibrator, while the oscillator that is composed of a head block, piezoelectric ceramics, and an end cap in sequence along the Y-direction is considered as a vertical rectangular vibrator, the stator can be regarded as two vertical rectangular vibrators and a horizontal rectangular vibrator intersecting, respectively. To explain the working principle, the side of the horizontal oscillator in the positive direction along Y-axis is indicated as the outer side, and vice versa, while the side of the vertical oscillator close to the driving feet is indicated as the outer side, and the side close to the clamping part as the inner side. For the working mode shown in Figure 3a, when the outer side of the horizontal oscillator contracts and the inner side stretches, the corresponding parts of the vertical oscillator act inversely, that is, the outer side of the vertical oscillator stretches and the inner side contracts. At this point, when the stator compresses the slider, a negative force along the Y-axis would be generated, which would drive the slider to move in the negative direction along the Y-axis. Thus, the inward motion is completed. Contrary to the above process, for the working mode shown in Figure 3b, when the outer side of the horizontal oscillator contracts and the inner side stretches, the corresponding parts of the vertical oscillator act simultaneously. In this case, when the stator compresses the slider, the force generated along the Y-axis points to the positive direction of the Y-axis, which would drive the slider to move in the positive direction along the Y-axis. Thus, the outward motion is accomplished.

Linear ultrasonic motors with dual driving feet can improve the vibration utilization efficiency of the vibrator, so as to improve the output performance of motor. Therefore, a lot of related research has been reported [28,29]. However, compared with most dual driving feet ultrasonic motors at the present stage, the relative position of the stator and slider of the Π-type motor is different. As presented in Figure 2 and Figure 3, if the connection line of two driving feet is the X-axis, for the Π-type motor, the slider is arranged on both sides of the stator and its motion direction will be along the Y-axis, which is perpendicular to the X-axis, while for other dual driving feet motors, the slider is usually arranged on one side of the stator, which is parallel to the X-axis, and its motion direction is also along the X-axis. According to the working principle of the linear ultrasonic motor, when the motor is running, the stator and the slider cannot be separated from each other macroscopically. For the case that the motor is required to accomplish a stroke of *s*, and the distance between the two driving feet is *d*, then for other motors, the length of slider needs to be at least s+d, while for Π-type motor, the length of slider can be *s*. That is to say, under the condition that a similar stroke is required to be accomplished, the length of slider needed by the Π-type motor is shorter, and the space utilization ratio is higher, while with a constant volume for motors, the Π-type motor can achieve a larger operation range.

### 2.2. Optimization of the Stator

According to the working principle of the Π-type motor, the trajectory of the driving foot is an inclined straight line, with the tangential component parallel to the contact interface between the stator and the slider and the normal component vertical to the contact interface. The normal component plays the role of making the driving foot contact or break away from the slider periodically and providing the dynamic preload between the stator and the slider, while the tangential component is used to drive the slider. Therefore, the amplitude of the driving foot has a significant influence on the output performance of the motor. Increasing the amplitude of the driving foot can drive the slider more effectively, which is conducive to improving the output performance of the motor.

**Figure 4:**
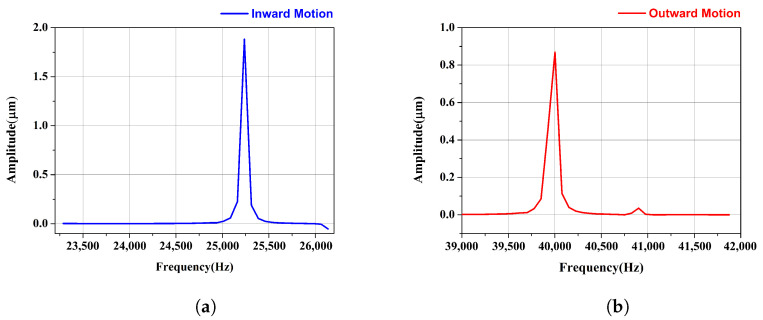
Amplitude of the driving foot of the original motor for (**a**) inward motion and (**b**) outward motion.

The working frequency of the original stator is obtained by modal analysis using ANSYS software (version 17.0). Further, the harmonic response analysis of the prototype stator is conducted, and the amplitudes of the driving foot are obtained, as shown in Figure 4. The working frequencies of the motor are 25,237 Hz for the inward motion and 40,001 Hz for the outward motion, respectively. When the driving voltage is 80 $V_{\mathrm{pp}}$, the corresponding amplitudes of the driving foot are 1.88 μm for the inward motion and 0.87 μm for the outward motion. According to previous experimental results, the amplitude for outward motion obtained by simulation is too small, and the motor may not achieve satisfactory output performance.

**Figure 5:**
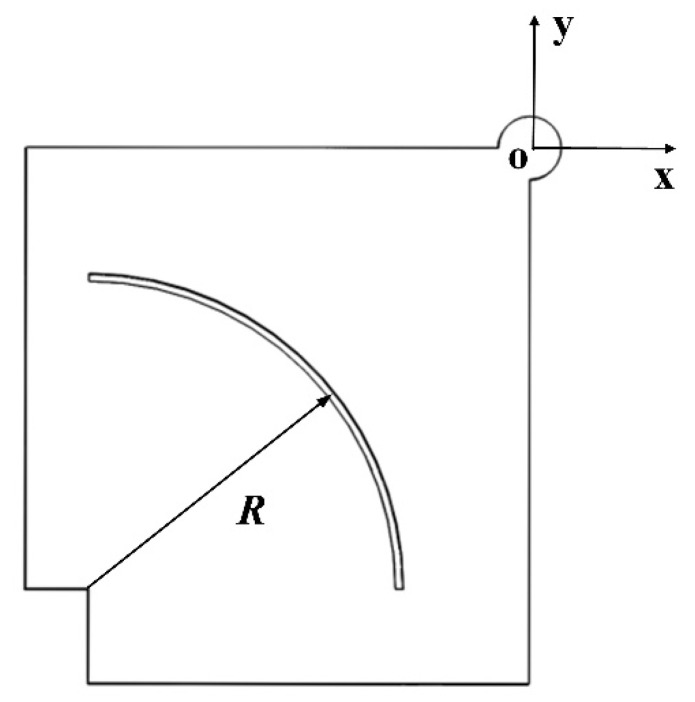
Head block of arc slotted stator.

Therefore, a slotting optimization scheme for head blocks is proposed. In order to reduce the stiffness of the head blocks and enlarge the amplitudes of the driving foot, an arc groove scheme is implemented, as presented in Figure 5.

**Figure 6:**
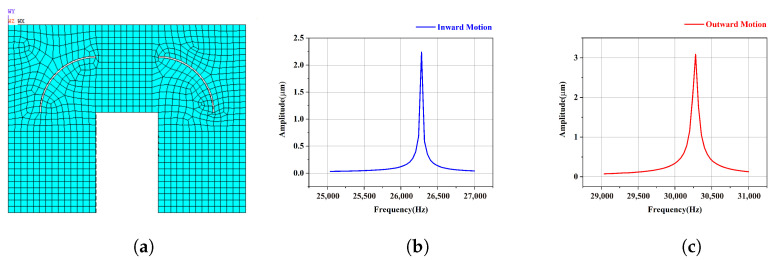
Amplitude of the driving foot of the arc slotted motor (**a**) for (**b**) inward motion and (**c**) outward motion.

If the driving foot center is taken as the origin and a coordinate system is established as shown in Figure 5, since the length of the piezoelectric ceramics used is 14 mm, the center of the arc groove is located at (−28, −28). Using the radius *R* of the arc groove as a parameter, a parameterized finite element model of the Π-type stator is established. The final value of *R* is determined to be 20 mm, and the width of the groove is 0.5 mm though simulation analysis. Modal analysis and harmonious response analysis are also carried out. The obtained working frequencies of the slotted motor are 26,280 Hz for the inward motion and 30,080 Hz for the outward motion, as shown in Figure 6, and the corresponding amplitudes of driving foot are 2.24 μm and 3.05 μm, respectively. For the inward motion, the amplitude of the driving foot is amplified to 1.19 times that of the original motor, while for the outward motion, the amplitude of the driving foot is amplified to 3.50 times that of the original motor, which proves that this slotting method is effective. In addition, for designing a novel ultrasonic motor, a lower order working mode is often chosen, because the motor can output more energy with a lower mode when the input energy is the same. Obviously, the working frequency for the outward motion decreases greatly after slotting, which also contributes to improving the output. In conclusion, this arc slotting method is expected to effectively improve the output performance of the motor.

**Figure 7:**
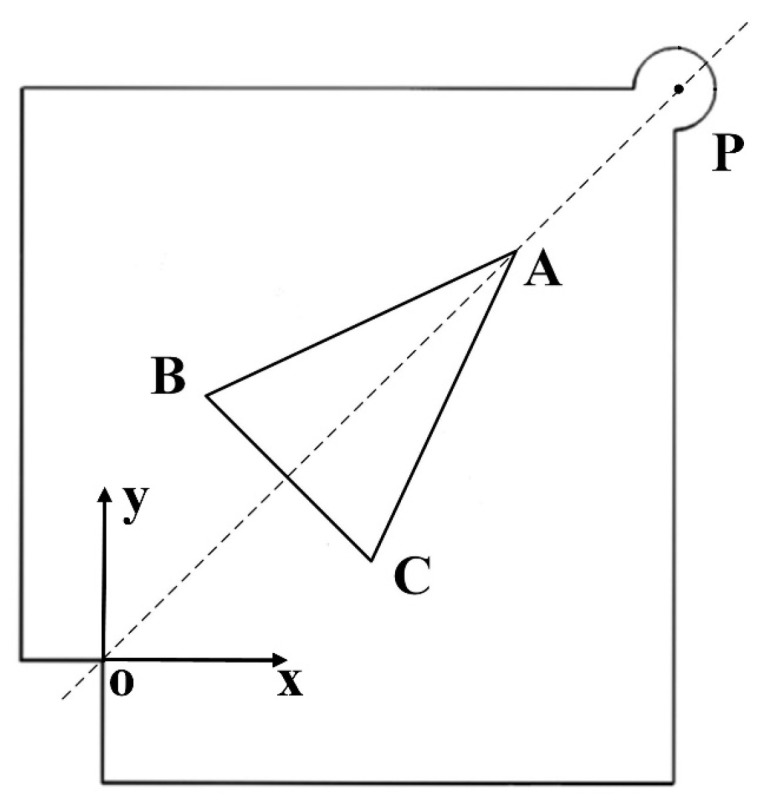
Head block of V-shaped slotted stator.

In References [30,31], another design method for ultrasonic motors’ head blocks is put forward. Using an amplitude transformer with continuous variable cross-section acts as the head block is considered to be beneficial to improving the wave propagation efficiency in the stator and enlarging the amplitude of the driving foot. Based on this, another slotting method is also proposed, as presented in Figure 7. This V-shaped slotting forms a variable section beam with continuous contraction of the cross-section between the end face of head block and the driving foot, that is, the end close to the piezoelectric ceramic is a large section and the end close to the driving foot is a small section. Similar to the function of an ultrasonic amplitude transformer, the amplitude of the driving foot can be amplified and the vibration speed can be increased by adopting this progressively shrinking variable of cross-section beam, and the energy can be concentrated on the driving foot to achieve energy accumulation.

If point O is taken as the origin and a coordinate system is established as shown in Figure 7, according to the dimension parameters of the Π-type stator, the coordinates of the driving foot center point P are (28, 28). Considering the symmetry of structure, the triangular groove is designed as an isosceles triangle, and the line OP is the bisector of the vertex angle. If the coordinates of the three vertices of the isosceles triangle are A($x_{a},y_{a}$), B($x_{b},y_{b}$), and C($x_{c},y_{c}$), respectively. It can be inferred from geometric relationships that $x_{a}=y_{a}$, $x_{b}=y_{c}$, and $x_{c}=y_{b}$. Therefore, the dimension of the slot can be determined by three parameters. Using ($x_{a},x_{b},x_{c}$) as parameters, a parameterized finite element model of the stator with the V-shaped slot is established. The range of the parameters is constrained based on the dimensions of the head block, the position, and the strength of the internal threaded hole. The final dimension parameters are determined through simulation analysis as $x_{a}$ = 24 mm, $x_{b}$ = 10 mm, and $x_{c}$ = 18 mm.

**Figure 8:**
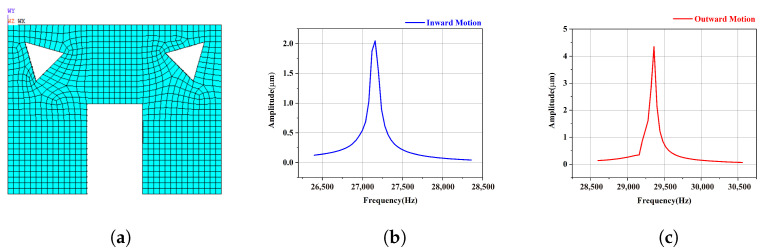
Amplitude of the driving foot of the V-shaped slotted motor(**a**) for (**b**) inward motion and (**c**) outward motion.

The modal analysis and harmonic response analysis of the slotted stator show that the working frequencies are 27,160 Hz for the inward motion and 29,360 Hz for the outward motion, as shown in Figure 8, and the corresponding amplitudes of the driving foot are 2.05 μm for the inward motion, 1.09 times that of the original motor, and 4.35 μm for the outward motion, 4.99 times that of the original motor, respectively. It is obvious that this V-shaped slotting is expected to dramatically improve the output for outward motion.

### 2.3. New Optimization Method for the Stator Based on Power Flow

The above optimization method for the stator is widely used in current research, that is, to optimize the stator structure with the aim of improving the vibration response (such as displacement response, velocity response, or stress response) of the stator. There are also optimization methods that aim at keeping away from interference modes or at the consistency of working modes’ frequencies [32,33,34]. However, these methods are only quantitative expressions of the stator’s vibration according to the magnitude of vibration response, rather than describing the nature of the vibration propagation in the stator. For the ultrasonic motor as a complex electromechanical coupling system, it is not enough to obtain the structural vibration response, but the transmission path of vibration energy in the stator is also needed. By analyzing the vibration transmission mechanism and the difference of vibration transmission among the stator components, we can judge whether the stator structure is reasonable and find out the components that need to be optimized and improved. Therefore, a new optimization method based on the power flow method is proposed. Specifically, for the purpose of making the most efficient use of the vibration energy of the stator, this method aims to convey as much vibration energy as possible to the contact interface of the stator and slider and convert it into the kinetic energy output of the slider.

At any point of the stator, a tiny regular hexahedron is taken as the research object. This element is affected by the elastomers around it, and the forces on each surface are expressed as one normal stress and two shear stress components. The differential equation for the motion of this element can be established as

(1) $$\left\{\begin{array}{l} \frac{\partial \sigma_{x}}{\partial x}+\frac{\partial \tau_{yx}}{\partial y}+\frac{\partial \tau_{zx}}{\partial z}+f_{x}=\rho \frac{\partial^{2}u}{\partial t^{2}}\\ \frac{\partial \tau_{xy}}{\partial x}+\frac{\partial \sigma_{y}}{\partial y}+\frac{\partial \tau_{zy}}{\partial z}+f_{y}=\rho \frac{\partial^{2}v}{\partial t^{2}}\\ \frac{\partial \tau_{xz}}{\partial x}+\frac{\partial \tau_{yz}}{\partial y}+\frac{\partial \sigma_{z}}{\partial z}+f_{z}=\rho \frac{\partial^{2}w}{\partial t^{2}} \end{array}\right.$$

By multiplying the two sides of the above three equations by u,v, and w, respectively, and adding them together, the simplified equation can be obtained as follows:

(2) $$\nabla \cdot \left(\sigma \cdot \frac{\partial S}{\partial t}\right)+F\cdot \frac{\partial S}{\partial t}-A=\frac{\partial }{\partial t}\left(\frac{\rho }{2}\frac{\partial S}{\partial t}\cdot \frac{\partial S}{\partial t}\right)$$

where ∇ is the Nabla operator and ρ is the material density. σ represents the stress matrix

(3) $$\sigma =\left[\begin{array}{ccc} \sigma_{x} & \tau_{xy} & \tau_{xz}\\ \tau_{yx} & \sigma_{y} & \tau_{yz}\\ \tau_{zx} & \tau_{zy} & \sigma_{z} \end{array}\right]$$

S={u,v,w} represents the displacement vector, $F=\{f_{x},f_{y},f_{z}\}$ represents the force vector, and *A* is expressed as

(4) $$A=\sigma_{x}\frac{\partial \dot{u}}{\partial x}+\sigma_{y}\frac{\partial \dot{v}}{\partial y}+\sigma_{z}\frac{\partial \dot{w}}{\partial z}+\tau_{xy}\left(\frac{\partial \dot{u}}{\partial y}+\frac{\partial \dot{v}}{\partial x}\right)+\tau_{yz}\left(\frac{\partial \dot{v}}{\partial z}+\frac{\partial \dot{w}}{\partial y}\right)+\tau_{zx}\left(\frac{\partial \dot{w}}{\partial x}+\frac{\partial \dot{u}}{\partial z}\right)$$

The energy of the element is composed of kinetic energy and potential energy. The unit volume energy is assumed to be

(5) $$e=e_{k}+e_{p}$$

where $e_{k}$ is kinetic energy density and $e_{p}$ is potential energy density, which can be expressed as

(6) $$e_{k}=\frac{\rho }{2}\frac{\partial S}{\partial t}\cdot \frac{\partial S}{\partial t}$$

(7) $$e_{p}=\frac{1}{2E}\left[{\sigma_{x}}^{2}+{\sigma_{y}}^{2}+{\sigma_{z}}^{2}-2\mu \left(\sigma_{x}\sigma_{y}+\sigma_{y}\sigma_{z}+\sigma_{z}\sigma_{x}\right)+2\left(1+\mu \right)\left({\tau_{xy}}^{2}+{\tau_{yz}}^{2}+{\tau_{zx}}^{2}\right)\right]$$

(8) $$\frac{\partial e_{p}}{\partial t}=A$$

Equation (2) can be rewritten as

(9) $$\frac{\partial e}{\partial t}+\nabla \cdot \left(-\sigma \cdot \frac{\partial S}{\partial t}\right)=F\cdot \frac{\partial S}{\partial t}$$

When the right end of Equation (9) is 0, the energy conservation formula of the element is obtained as

(10) $$\frac{\partial e}{\partial t}+\nabla \cdot \left(-\sigma \cdot \frac{\partial S}{\partial t}\right)=0$$

Let

(11) $$i=-\sigma \cdot \frac{\partial S}{\partial t}$$

$i=\{i_{x},i_{y},i_{z}\}$ is called the transient structural intensity vector. It can be seen from Equation (11) that it represents the flow direction and distribution of energy at any point of the structure in the vibration process. The components of i along the three coordinate directions can be deduced to be

(12) $$\left\{\begin{array}{c} i_{x}\left(t\right)=-\left(\sigma_{x}\dot{u}+\tau_{xy}\dot{v}+\tau_{xz}\dot{w}\right)\\ i_{y}\left(t\right)=-\left(\tau_{yx}\dot{u}+\sigma_{y}\dot{v}+\tau_{yz}\dot{w}\right)\\ i_{z}\left(t\right)=-\left(\tau_{zx}\dot{u}+\tau_{zy}\dot{v}+\sigma_{z}\dot{w}\right) \end{array}\right.$$

If the time average of the transient structural intensity’s component in k direction is recorded as $I_{k}=\langle$$i_{k}\left(t\right)$〉, then

(13) $$I_{k}=\langle i_{k}\left(t\right)\rangle =\left(1/T\right)\int_{0}^{T}i_{k}\left(\tau \right)d\tau$$

For the steady state vibration excited by a single frequency, the transient structural intensity in each direction after a time average can be expressed by a complex number [35,36] as

(14) Ixω=ix(t)=−1/2Re(σ˜xu˙˜*+τ˜xyv˙˜*+τ˜xzw˙˜*)Iyω=iy(t)=−1/2Re(τ˜yxu˙˜*+σ˜yv˙˜*+τ˜yzw˙˜*)Izω=iy(t)=−1/2Re(τ˜zxu˙˜*+τ˜zyv˙˜*+σ˜zw˙˜*)

where σ, τ represents complex stress, u*, v*, and w* represent conjugates of complex velocity, ω = 2πf represents the circular frequency of the excitation signal, f represents the working frequency of the stator, and Re is the real part of complex number.

For steady state vibration, the following relations are established for each direction of vibration:

(15) $$\left\{\begin{array}{c} \tilde{\dot{u}}=\omega j\cdot \tilde{u}\\ \tilde{\dot{v}}=\omega j\cdot \tilde{v}\\ \tilde{\dot{w}}=\omega j\cdot \tilde{w} \end{array}\right.$$

Here, *u*, *v*, and *w* denote complex displacement and j denotes the imaginary unit.

By substituting Equation (15) into Equation (14), the structural intensity of the steady state vibration in three directions can be expressed by stress and displacement parameters as

(16) Ixf=−πfIm(σ˜xu˜*+τ˜xyv˜*+τ˜xzw˜*)Iyf=−πfIm(τ˜yxu˜*+σ˜yv˜*+τ˜yzw˜*)Izf=−πfIm(τ˜zxu˜*+τ˜zyv˜*+σ˜zw˜*)

The working frequency of the stator can be obtained by ANSYS modal analysis. Then, the harmonic response of the stator is conducted around the working frequency to obtain the geometrical relationship, complex stress, and complex displacement. Importing this information into Matlab software (version 2018), the time-averaged structural intensity vector can be calculated according to Equation (16). Finally, the visualization of structural intensity can be realized by programming.

**Figure 9:**
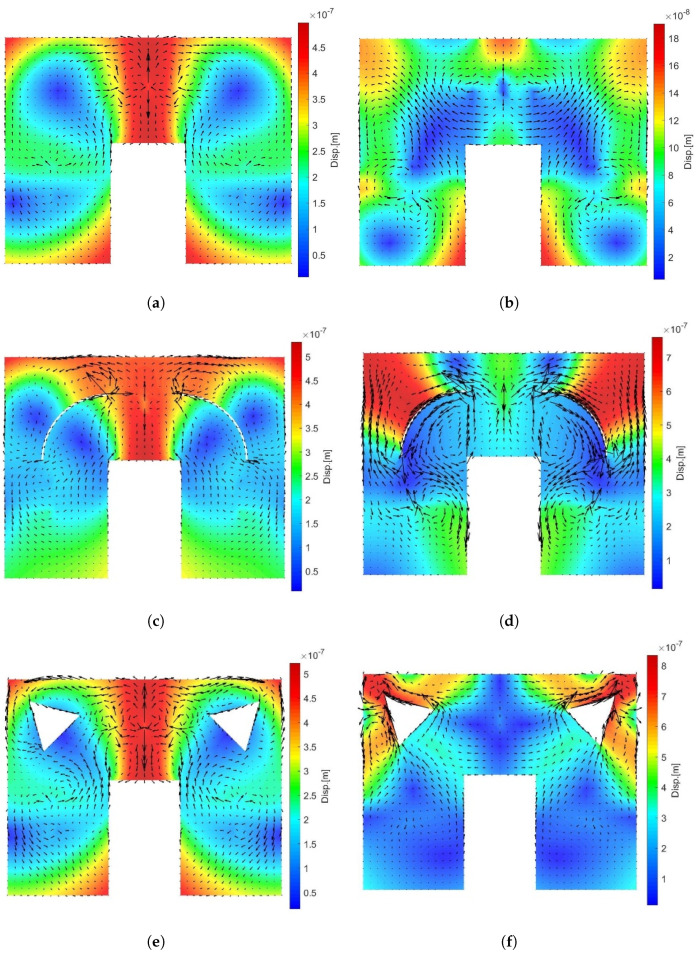
Visualization of the structural intensity of (**a**) the original motor for inward motion, (**b**) the original motor for outward motion, (**c**) the arc slotted motor for inward motion, (**d**) the arc slotted motor for outward motion, (**e**) the V-shaped slotted motor for inward motion, and (**f**) the V-shaped slotted motor for outward motion.

The above method is used to visualize the structural intensity of the three stators, and the results are shown in Figure 9. The arrow in the figure represents the structural intensity vector of the stator in the plane xoy, and the cloud image is the distribution of the vibration displacement field of the stator.

Comparing the energy transmission paths of the three motors for the inward motion and the outward motion, the influence of slotting on two working modes can be obtained, so whether the slotting method is reasonable can be ascertained. For the original motor without slotting, the structural intensity and displacement field of the inward motion are shown in Figure 9a. It can be seen that the displacement of the driving foot is large and the direction of energy flow is reasonable. However, in the horizontal oscillator, most of the energy generated by the piezoelectric ceramics still remains in the piezoelectric ceramic part, only a small part of which flows to both driving feet, while in the vertical oscillator, part of the energy flows to the driving feet, and the other part of the energy forms a closed energy eddy current field in the middle of the oscillator. The existence of the eddy current field indicates the conservation of energy inflow and outflow in this region. After an arc slot is cut at the head block of the stator, as presented in Figure 9c, the energy generated in the piezoelectric ceramics of the horizontal oscillator is no longer concentrated in the piezoelectric ceramics, but is released in large quantities and flows to the driving feet on both sides. Although the eddy current field still exists in the vertical oscillator, some energy also flows to the driving foot. For the stator with the V-shaped slot shown in Figure 9e, the energy of the horizontal oscillator is also released. Compared with the former two stators, this V-shaped slot leads to energy flow in the vertical oscillator. Although the energy eddy current field still exists, the energy of the vertical oscillator’s inner part is released and flows to the driving feet along the notch. In general, the two slotting methods can slightly amplify the displacement response of the driving foot, effectively release the energy of piezoelectric ceramics to flow to the driving foot, and lead the overall energy flow characteristics of motor to be more reasonable, which is conducive to improving the output performance of motor.

Similarly, the outward motions are analyzed. For the original stator shown in Figure 9b, the amplitude of the driving foot is very small, and the flow of energy is chaotic, with several energy eddy fields. After the stator is slotted, the working mode is improved effectively, as presented in Figure 9d. The amplitude of the driving foot is dramatically amplified, and most of the energy is concentrated towards the driving foot. However, part of the energy is wasted as a distinct eddy current field forms below the arc groove. This situation is changed for the V-shaped slotted stator, as illustrated in Figure 9f. Although the eddy current still exists in the variable cross-section beam part of the vertical oscillator, most of the stator’s energy flows to the driving foot, forming an effective energy aggregation, and the displacement response of the driving foot is much larger than that of the other parts. In conclusion, the outward motion of the original stator is obviously poor, and either the displacement response or the energy flow is unreasonable, which cannot meet the application requirements of ultrasonic motors. Both slotting methods can effectively amplify the amplitude of the driving foot and lead the energy flow in the stator to be more reasonable. Especially for the V-shaped slotted stator, the variable cross-section beam not only dramatically enlarges the displacement response of the driving foot, but also concentrates energy to the driving foot. Compared with the inward motion, the slotting optimization method may have a more obvious impact on the outward motion, which is expected to significantly improve the output performance of motor for the outward motion.

## 3. Experimental Investigation

**Figure 10:**
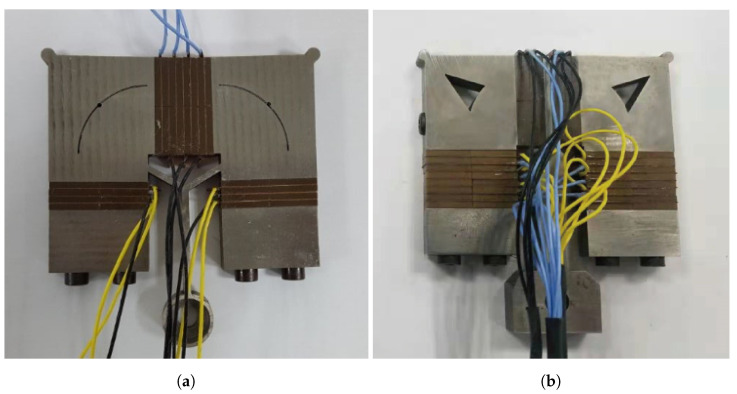
Prototypes for (**a**) the arc slotted stator and (**b**) the V-shaped slotted stator.

In order to verify the output performance of the novel motors, the prototypes were manufactured, as shown in Figure 10.

First of all, the working frequencies of the two slotted stators were measured by a laser vibrometer (PSV-300F-B0, Polytec, Waldbronn, Germany). For the arc slotted stator, the working frequencies obtained are 26,654 Hz for the inward motion and 30,679 Hz for the outward motion, respectively. Similarly, the working frequencies of the V-shaped slotted stator are 27,587 Hz for the inward motion and 29,698 Hz for the outward motion. This is different from the simulation results. These differences are mainly due to the differences between the material parameters used in the simulation process and the actual material parameters, as well as the errors caused by the processing and assembly.

**Figure 11:**
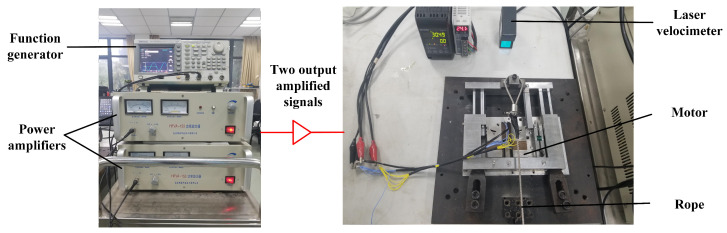
Experimental setup.

In order to test the output characteristics of the two stators, experiments were performed with a special set up presented in Figure 11. A signal generator (AFG3022B, Tektronix, Beaverton, OR, USA) and two high frequency amplifiers (HFVP-153, Foneng, Nanjing, China) were utilized as the driving power source. When the slider was driven by the stator, a laser velocimeter was adopted to record the speed of the slider and send it to a computer. The output speed of the motor is measured for various preloads and driving frequencies. In order to obtain the output thrust, a fixed pulley is installed on the test platform. A rope is used to connect the slider and the weights, and the latter are considered to be equal to the output thrust.

**Figure 12:**
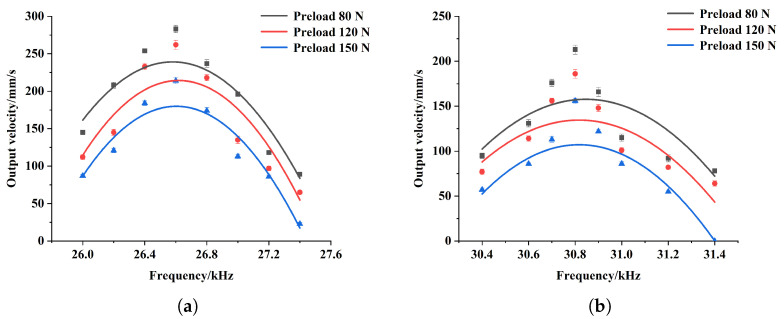
Relation between output velocity and excitation frequency of the arc slotted stator for inward motion (**a**) and outward motion (**b**) at various preload levels.

Firstly, the output performance of the arc slotted stator was tested. The prototype was placed in the above test system, and an excitation voltage of 500 $V_{\mathrm{pp}}$ was applied to it. The no-load velocity of the prototype was measured by the laser velocimeter. The driving principle of the linear ultrasonic motor satisfies the Coulomb friction model, which means that the preload to some extent determines the output thrust of the motor. In order to obtain a large output thrust, it is necessary to apply a large preload. However, excessive preload can suppress the vibration of the stator, causing a decrease in the output thrust and even causing the motor to stop running. As for the output velocity, it usually decreases with the increase in preload. The preliminary experimental results show that the Π-type motor can operate within a preload range of 80 N to 190 N [25]. Taking both output thrust and velocity into account, three preload values of 80 N, 120 N, and 150 N were selected as experimental values within this range. Springs with different stiffness were put into the preload device, so that the preload of the motor reached 80 N, 120 N, and 150 N, respectively. Under these three preloads, the relationship between the no-load velocity and excitation frequency was obtained, as shown in Figure 12.

In general, regardless of the motion direction, the output velocity is influenced by both the driving frequency and the preload. Specifically, when the preload remains unchanged, the output velocity of the motor first increases and then decreases with the increase in excitation frequency, and reaches a peak value around the working frequency. When the excitation frequency remains constant, the output velocity decreases with the increase in preload. For the inward motion, the motor can effectively operate when the excitation frequency is between 26 kHz and 27.4 kHz, and reaches the maximum output velocity of 283 mm/s when the excitation frequency is 26.6 kHz and the preload is 80 N. Meanwhile, for the outward motion, the effective excitation frequency bandwidth of the motor is from 30.4 kHz to 31.4 kHz, and the motor reaches the maximum output velocity of 213 mm/s when the excitation frequency is 30.8 kHz and the preload is 80 N.

**Figure 13:**
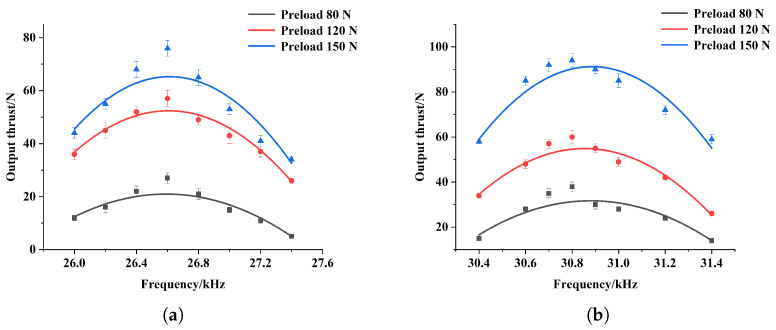
Relation between output thrust and excitation frequency of the arc slotted stator for inward motion (**a**) and outward motion (**b**) at various preload levels.

The preloads of 80 N/120 N/150 N were still selected to measure the output thrust of the motor. The relationship between the output thrust and the excitation frequency is presented in Figure 13. It can be seen that the output thrust is also affected by both the excitation frequency and the preload. When the preload remains unchanged, the output thrust of the motor first increases and then decreases with the increase in excitation frequency, and reaches the peak value around the working frequency. When the excitation frequency remains constant, the output thrust increases with the increase in preload. In addition, compared with the output velocity, the output thrust is more significantly affected by the preload. For applications requiring a certain output thrust, an appropriate range of preload is particularly important. This is because if the preload is too small, the motor cannot output a large thrust, regardless of the excitation frequency. For this arc slotted motor, the maximum output thrust is 76 N for the inward motion with the excitation frequency of 26.6 kHz and a preload of 150 N, while for the outward motion, the maximum output thrust is 94 N when the excitation frequency is 30.8 kHz and the preload is 150 N.

**Figure 14:**
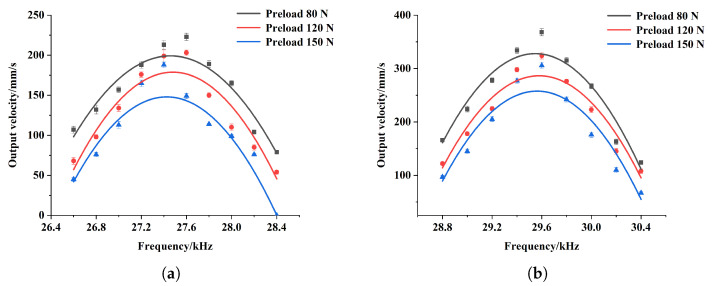
Relation between output velocity and excitation frequency of the V-shaped slotted stator for inward motion (**a**) and outward motion (**b**) at various preload levels.

**Figure 15:**
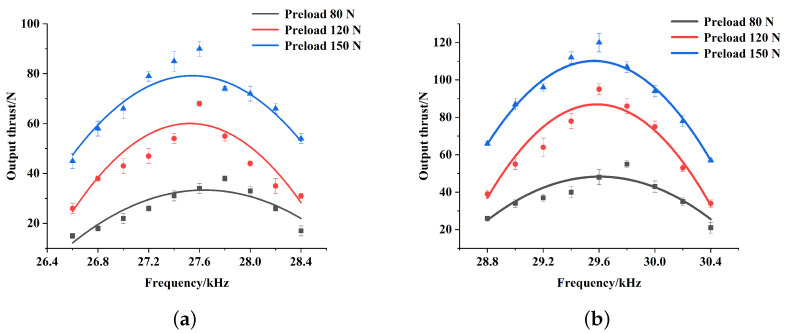
Relation between output thrust and excitation frequency of the V-shaped slotted stator for inward motion (**a**) and outward motion (**b**) at various preload levels.

The output characteristics of the V-shaped slotted motor were verified with the same experimental conditions and methods. The relationship between the output velocity and the excitation frequency, as well as the relationship between the output thrust and the excitation frequency under a certain preload was obtained and illustrated in Figure 14 and Figure 15.

Apparently, on the whole, the influence of preload and excitation frequency on the output performance of the V-shaped slotted motor is consistent with that of the arc slotted motor. The difference is that, for the V-shaped slotted motor, when the excitation frequency of inward motion is between 26.5 kHz and 28.4 kHz and the excitation frequency of outward motion is between 28.8 kHz and 30.4 kHz, the motor can operate stably. That is to say, the excitation frequency bandwidth of this motor is wider and its adjustability is better. Specifically, when the excitation frequency is 27.6 kHz and the preload is 80 N, the maximum output velocity for inward motion is 223 mm/s, and when the excitation frequency is 29.6 kHz and the preload is 80 N, the maximum output velocity for outward motion is 368 mm/s. As far as the output thrust is concerned, the maximum output thrust for inward motion reaches 90 N with an excitation frequency of 27.6 kHz and a preload of 150 N, while the maximum output thrust for outward motion reaches 120 N with an excitation frequency of 29.6 kHz and a preload of 150 N. Moreover, compared with the arc slotted motor, the output performance for the outward motion has been dramatically improved. This is consistent with the simulation results, which shows that the new optimization method is feasible and effective. It can predict the performance and optimize the structure of the motor from the point of view of power flow.

In conclusion, both new motors can achieve excellent output performance, and their output performance is affected by the synergistic effect of the excitation frequency and the preload. According to the specific requirements and technical indicators of the application occasions, the output characteristics of the motor can be controlled by adjusting the preload and the excitation frequency.

## 4. Conclusions

In this paper, focusing on reducing the motor’s volume and improving its output characteristics, the Π-type linear ultrasonic motor has been studied. Firstly, the configuration and operating principle of a Π-type linear ultrasonic motor is illustrated. Secondly, two slotting schemes are proposed for the stator to reduce the stiffness of head blocks and enlarge the amplitude of driving foot, so as to improve the output characteristics of the motor. After that, a novel optimization method for ultrasonic motors is suggested. Based on the power flow principle, this new optimization method can judge whether the structure of the stator is reasonable by visualizing the energy flow characteristics of the stator. Finally, the output characteristics of the two new stators are verified by experiments. The experimental results demonstrate that the two new stators can both achieve excellent output performance. For the arc slotted stator, the motions in both directions are relatively close. The maximum output thrust is 76 N/94 N, and the corresponding maximum output velocity is 283 mm/s/213 mm/s. As for the V-type slotted stator, its outward motion performance is dramatically improved, with a maximum output thrust of up to 120 N and a maximum output speed of up to 368 mm/s, while the performance in the other direction also remains good, with a maximum output thrust of up to 90 N and a maximum output speed of up to 223 mm/s. The experimental results are consistent with the predictions of simulation analysis, indicating that the optimization method based on power flow is effective. In summary, these two novel motors can efficiently output a large thrust in a narrow space.

## Figures and Tables

**Table 1:** Material parameters of the Π-type motor.

Materials	PZT-8	Cr12	${\mathrm{Al}}_{2}$ $O_{3}$
Dimension (${\mathrm{mm}}^{3}$)	14 × 8 × 2	/	100 × 10 × 3
Density (kg/$m^{3}$)	7650	7850	3920
Poisson’s ratio	0.31	0.28	0.22
Young’s modulus (×$10^{10}$ N/$m^{2}$)	$\left(\begin{array}{cccccc} 12.06 & 5.35 & 5.15 & 0 & 0 & 0\\ 5.35 & 12.06 & 5.15 & 0 & 0 & 0\\ 5.15 & 5.15 & 10.45 & 0 & 0 & 0\\ 0 & 0 & 0 & 3.13 & 0 & 0\\ 0 & 0 & 0 & 0 & 3.13 & 0\\ 0 & 0 & 0 & 0 & 0 & 3.46 \end{array}\right)$	21.8	34
Piezoelectric constant (C/$m^{2}$)	$\left(\begin{array}{ccc} 0 & 0 & -5.2\\ 0 & 0 & -5.2\\ 0 & 0 & 15.1\\ 0 & 12.7 & 0\\ 12.7 & 0 & 0\\ 0 & 0 & 0 \end{array}\right)$	/	/

## Data Availability

The original contributions presented in the study are included in the article, further inquiries can be directed to the corresponding author.
